# Rimklb mutation causes male infertility in mice

**DOI:** 10.1038/s41598-021-84105-z

**Published:** 2021-02-25

**Authors:** Koji Maekura, Satoshi Tsukamoto, Michiko Hamada-Kanazawa, Masaoki Takano

**Affiliations:** 1 Laboratory of Molecular Cellular Biology, School of Pharmaceutical Sciences, Kobe Gakuin University, 1-1-3 Minatojima, Chuo-ku, Kobe, 650-8586 Japan; 2 Laboratory Animal and Genome Sciences Section, National Institute for Quantum and Radiological Science and Technology, 4-9-1 Anagawa, Inage-ku, Chiba, 263-8555 Japan

**Keywords:** Spermatogenesis, Germline development

## Abstract

Rimklb is a mammalian homologue of the *E. coli* enzyme RimK, which catalyzes addition of glutamic acid to the ribosomal protein S6. To date, no previous studies have shown any physiological role for Rimklb in mammals. In this study, using Western blotting, we found that Rimklb is distributed and expressed in mouse testis and heart. Rimklb was subsequently localized to the testicular Leydig cells using immunohistochemistry with an anti-Rimklb antibody. We generated a Rimklb mutant mouse in which a three-base deletion results in deletion of Ala 29 and substitution of Leu 30 with Val, which we named the Rimklb^A29del, L30V^ mutant mouse. Rimklb^A29del, L30V^ mutant mice show a decrease in testicular size and weight, and in vitro fertilization demonstrates complete male infertility. Furthermore, we found that a key factor in the mammalian target of the rapamycin/ribosomal protein S6 transcriptional pathway is hyperphosphorylated in the seminiferous tubules of the mutant testis. We conclude that Rimklb has important roles that include spermatogenesis in seminiferous tubules. In summary, male Rimklb^A29del, L30V^ mice are infertile.

## Introduction

RimK is a unique protein, which in *Escherichia coli* acts as an enzyme that post-translationally modulates ribosomal protein S6 (S6)^1^. Bacterial S6 is a target for oligo-glutamylation by the ATP-dependent glutamate ligase RimK^2^. Post-translational modification of S6 involves addition of a glutamic acid residue to the C-terminus to regulate ribosomal function^3^. In *Pseudomonas aeruginosa*, deficiency of RimK affects its survival, toxicity, and plant infectivity, due to the functional effects of RimK on ribosomal properties^4^.

Rimklb is a mammalian homologue of RimK, and cDNA had been cloned in mammals^5^ resulting in a β-citrylglutamate (β-CG) or *N*-Acetylaspartylglutamate synthase that adds glutamic acid to substrates^6^. However, the physiological functions of Rimklb in mammals are still unknown, and this has not been studied previously.

The mammalian target of rapamycin (mTOR) plays a critical role in spermatogenesis^7^, and treatment with rapamycin suppresses mTOR, reducing sperm count and proliferation of spermatogonia^8^. Binding to Raptor, mTOR forms the mTORC1 complex that activates mammalian S6 protein kinase (S6K) and the S6 protein, using phosphorylation to regulate blood-testis barrier (BTB) dynamics^9,10^. Studies of animal models have shown that overexpression and phosphorylation of S6 disrupts the BTB and results in defective spermatogenesis, indicating that S6 plays an important role in BTB dynamics, regulating spermatogenesis^11^. These lines of study have shown that the mTORC/p70s6k/S6 pathway plays a crucial role in spermatogenesis.

To elucidate the physiological function of Rimklb in mammals, we generated Rimklb mutant mice that show a three-base deletion in exon 2 of the Rimklb gene, described as Rimklb^A29del, L30V^. We observed that Rimklb^A29del, L30V^ mice clearly show male infertility: testicular size and weight are reduced, resulting in a reduction in the success rate of in vitro fertilization. Our results suggested that Rimklb plays an essential role in spermatogenesis in mice.

## Results

### Expression of Rimklb in organs and tissues

**Figure 1:**
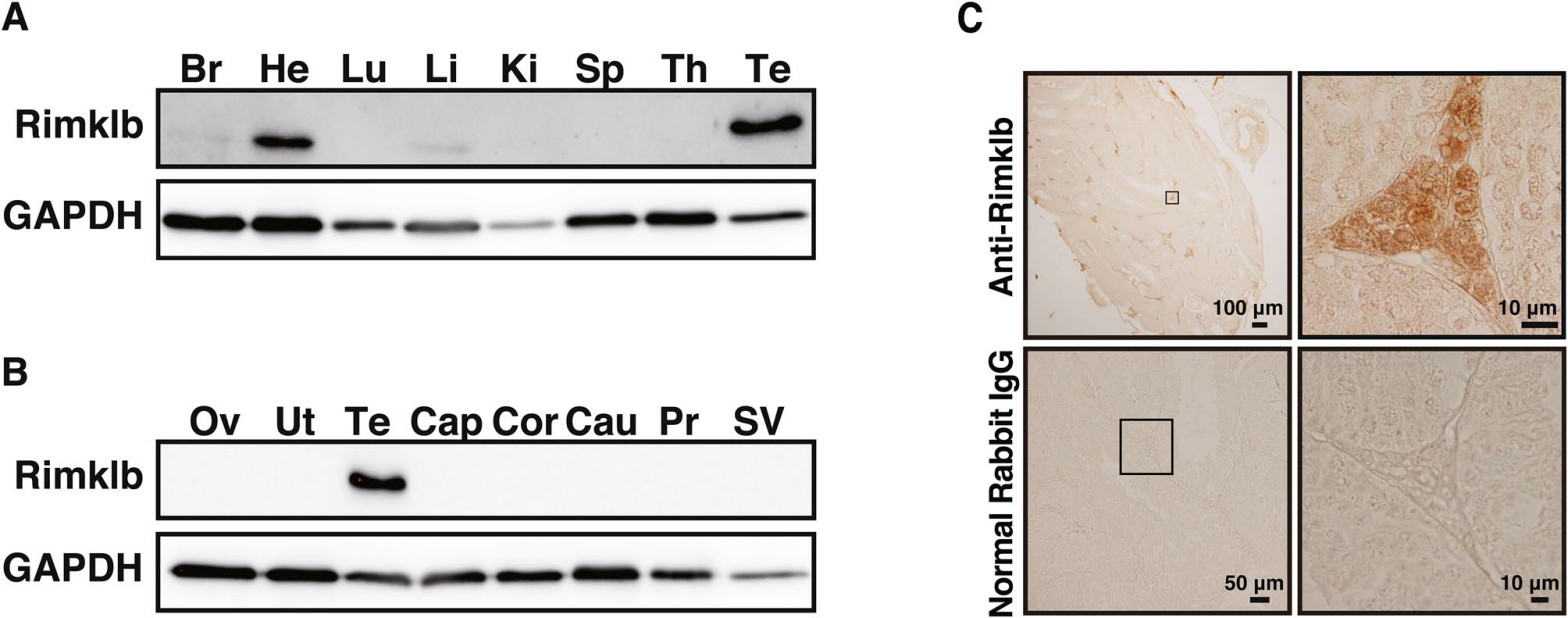
Expression profile of Rimklb in mouse tissues. (**A**) Western blot analysis of Rimklb. *Br* brain, *He* heart, *Lu* lung, *Li* liver, *Ki* kidney, *Sp* spleen, *Th* thymus, *Te* testis, (**B**) Western blot analysis of Rimklb in reproductive organs. *Ov* ovary, *Ut* uterus, *Cap* caput epididymidis, *Cor* corpus epididymidis, *Cau* cauda epididymidis, *Pr* prostate gland, *SV* seminal vesicle. GAPDH was used as a loading control. (**C**) Rimklb expression in mouse testis sections as determined by immunostaining, using normal rabbit IgG as a negative control. Enlarged images of the boxed area are shown (Right). Note that Rimklb is mainly expressed in the Leydig cells of the testis.

The expression of Rimklb in various organs of adult mice was analyzed by Western blotting. As shown in Fig. 1A, Rimklb was robustly expressed in heart and testis, and slightly expressed in brain and liver, which is consistent with a previous study where Rimklb was extracted from testes^5^. Moreover, we analyzed the expression pattern of Rimklb in various reproductive organs, and Rimklb was only expressed in the testis, with no expression in organs such as the ovary, uterus, epididymis (caput, corpus or cauda), prostate or seminal vesicle (Fig. 1B). Furthermore, immunohistochemical analysis revealed that Rimklb was mainly expressed in the Leydig cells of the testis (Fig. 1C).

### Generation of Rimklb^A29del, L30V^ mutant mice

**Figure 2:**
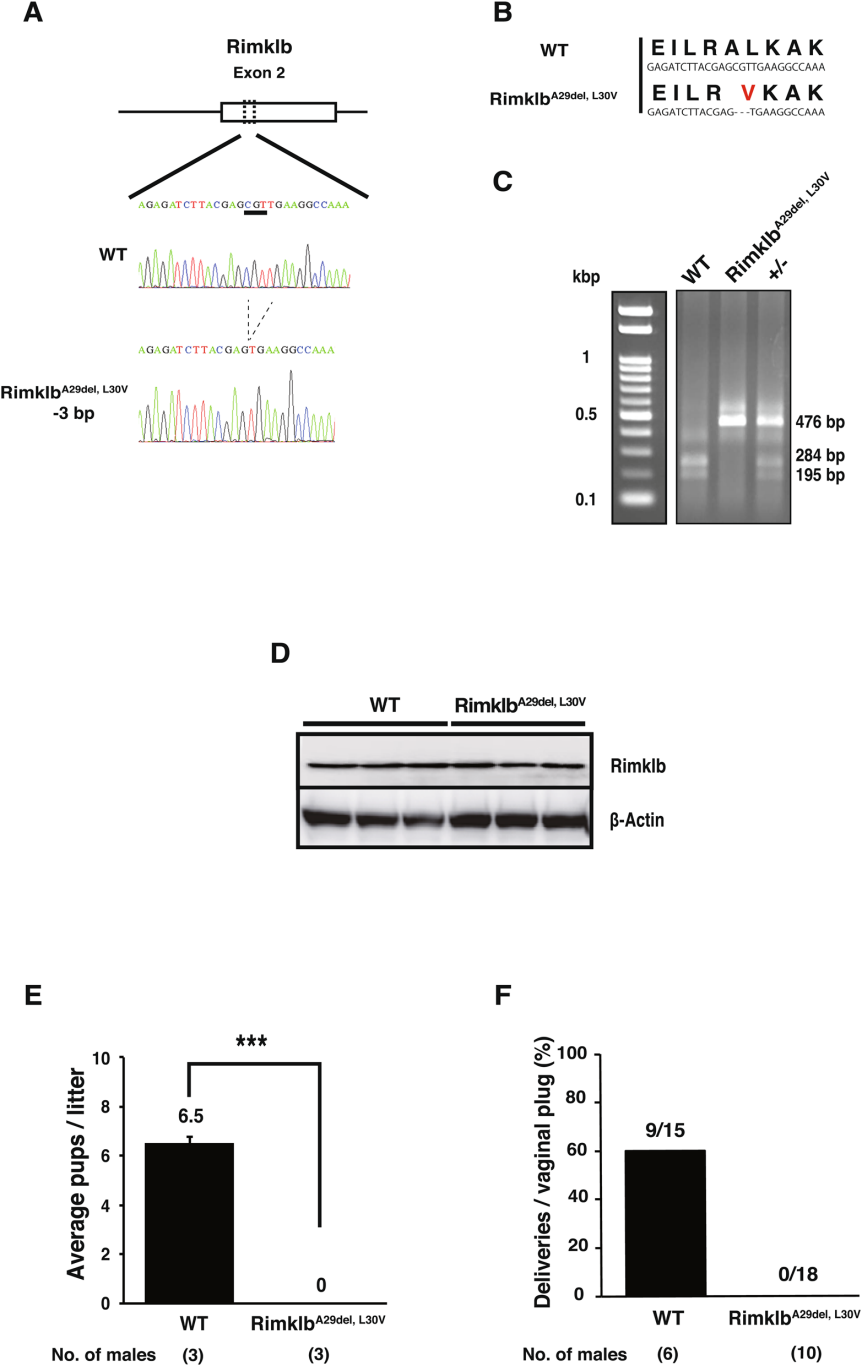
Rimklb^A29del, L30V^ mutant mice are infertile. (**A**) Diagram illustrating the Rimklb^A29del, L30V^ gene. Rimklb^A29del, L30V^ mice had three bases deleted in exon 2 (c.351_353del). (**B**) The deletion of 3 bp altered the Rimklb ORF. The amino acid sequence corresponding to the codons (DNA sequence) is shown in smaller letters below. (**C**) Genotyping of WT, Rimklb^A29del, L30V^. The electrophoretic image of the PCR product after Mwo I digestion. (**D**) Western blot analysis of Rimklb in WT and Rimklb^A29del, L30V^ mouse testis at age 12 weeks. β-Actin was used as a sample processing control. (**E**) The average number of pups per litter. Data are presented as mean ± standard error of the mean (SEM); Student’s t-test; ***p < 0.001. (**F**) Number of deliveries after vaginal plug formation.

To examine the physiological roles of Rimklb in vivo, we generated Rimklb mutant mice using a CRISPR/Cas9-mediated genome-editing approach. Using this approach, we obtained three lines of homozygous mutant mice, including two male mice and one female mouse. The DNA sequence obtained from the mutant mouse is shown as an electrophoretogram indicating a three-base deletion mutation (Fig. 2A). The deletion of three bases results in deletion of Ala 29 and substitution of Leu 30 with Val 30 (Fig. 2B); we call these Rimklb^A29del, L30V^ mutant mice. Potential off-target sites were identified using Off-spotter (https://cm.jefferson.edu) and CHOPCHOP (https://chopchop.cbu.uib.no). There were no genomic DNA sequences that differed from the Rimklb target site in one or two locations. Three sites with high similarity were selected and the nucleotide sequence was analyzed by direct sequencing; there were no deletions or insertions at these sites (Supplementary Fig. S1).

Genotyping was performed by PCR with associated use of the restriction enzyme Mwo I (Fig. 2C): PCR products from wild mice were cut by Mwo I, but the amplicon from mutant mice was not digested (Fig. 2C). On analyzing the expression of Rimklb protein, in which the signals were not different between mutant and wild-type mice testes (Fig. 2D, Supplementary Fig. S2A), the mutated Rimklb protein was assumed to be the same size as the wild type protein due to the single amino acid deletion of Ala 29 and the substitution of Leu 30 with Val 30.

### Rimklb^A29del, L30V^ mutation causes male infertility in mice

**Figure 3:**
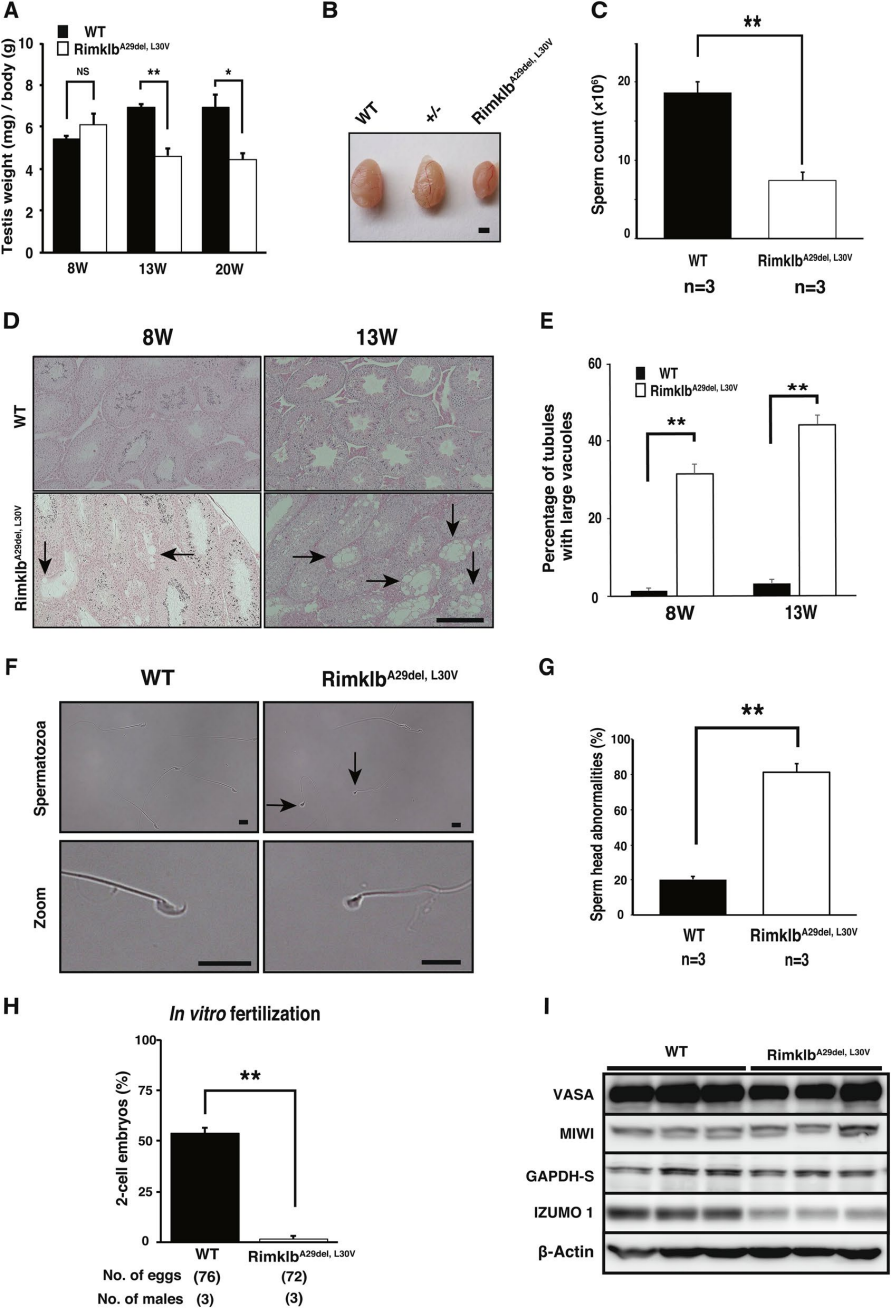
Rimklb^A29del, L30V^ mice show abnormal spermatogenesis. (**A**) Average testis/body weights of WT and Rimklb^A29del, L30V^ at 8, 13 and 20 weeks of age. Data are presented as mean ± SEM; Student’s t-test; **p < 0.01, *p < 0.05, *NS* not significant. (n = 3) (**B**) Representative images of WT and Rimklb^A29del, L30V^ testis at 20 weeks of age. Scale bar = 1 mm. (**C**) Average sperm counts of WT and Rimklb^A29del, L30V^ at 13 weeks of age. Data are presented as mean ± SEM; Student’s t-test; **p < 0.01. (**D**) Hematoxylin and eosin staining of tissue from WT (upper) and Rimklb^A29del, L30V^ (bottom) mice at 8 and 13 weeks of age. Vacuolated tubules in the testis are indicated by black arrows. Scale bar = 250 μm. (**E**) Percentage of tubules with large vacuoles. We scored 59–212 tubules from each animal. Data are presented as mean ± SEM; Student’s t-test; **p < 0.01. (n = 3) (**F**) Morphology of spermatozoa from WT (upper, left) and Rimklb^A29del, L30V^ (upper, right). The black arrows indicate sperm with abnormal head. Enlarged image of WT and Rimklb^A29del, L30V^ spermatozoal heads (bottom). Scale bar = 10 μm. (**G**) Cauda epididymidis sperm head abnormality ratio for WT and Rimklb^A29del, L30V^ mice. Data are presented as mean ± SEM; Student’s t-test; **p < 0.01 (**H**) In vitro fertilization rate with WT and Rimklb^A29del, L30V^ spermatozoa. The percentage of two-cell embryos 24 h after in vitro fertilization. Data are presented as mean ± SEM; Student’s t-test; **p < 0.01 (**I**) Western blot analysis of VASA, MIWI, GAPDH-S and IZUMO1 in WT and Rimklb^A29del, L30V^ mouse testes at 12 weeks of age. β-Actin was used as a sample processing control.

We tested the fertility of Rimklb^A29del, L30V^ male mice by mating them with wild C57BL/6 females for a two-month period (from eight weeks to 16 weeks of age). As shown in Fig. 2E,F, female mice showed plugs after mating with Rimklb^A29del, L30V^ male mice, but did not become pregnant and did not have pups, compared with the 60% litter rate per plug after mating with wild C57BL/6 male mice. These results indicate that Rimklb^A29del, L30V^ male mice were able to mate but were completely infertile. In addition, the weights of testes from Rimklb^A29del, L30V^ male mice were significantly reduced compared with wild C57BL/6 mice at age 13 or 20 weeks (Fig. 3A,B). Sperm counts were obviously decreased (Fig. 3C), and attenuation of sperm motility was observed in Rimklb^A29del, L30V^ male mice (Supplementary Video 1). There was no significant change in testosterone levels between wild type (WT) and Rimklb^A29del, L30V^ male mice (Supplementary Fig. S2B).

Histological analysis revealed large vacuoles in seminiferous tubules in the testes of eight-week-old Rimklb^A29del, L30V^ mice, which became prominent at 13 weeks (Fig. 3D), and the incidence of seminiferous tubules with large vacuoles was markedly increased in seminiferous tubules of Rimklb^A29del, L30V^ male mice at both 8 and 13 weeks (Fig. 3E). These results suggest that incomplete spermatogenesis occurs in testes of Rimklb^A29del, L30V^ male mice. Morphological evaluation of Rimklb^A29del, L30V^ sperm shows an abnormal head and a marked increase in the percentage of sperm head abnormalities, compared with the wild type (Fig. 3F,G). To evaluate sperm fertility, we performed in vitro fertilization (IVF) using the spermatozoa of three-month-old male mice, and further analysis revealed that Rimklb^A29del, L30V^ spermatozoa showed no fertility with intact oocytes; 53.8 ± 2.6% fertilized eggs were observed when oocytes were treated with wild type spermatozoa, whereas 1.7 ± 1.7% fertilized eggs were observed when oocytes were treated with Rimklb^A29del, L30V^ spermatozoa (Fig. 3H). We were able to observe Rimklb^A29del, L30V^ spermatozoa binding to the zona pellucida (ZP), and some eggs showed two pronuclei, 6 h after insemination (Supplementary Fig. S2C). These results suggest that the attenuation in the fertilization rate of oocytes is probably caused by multiple factors such as decreased motility and abnormal morphology of the sperm head. During spermatogenesis, some sperm-specific proteins are expressed. In the mutant Rimklb mouse testis we found reduced IZUMO1, a protein that is well known to play a role in sperm-egg fusion. Conversely, the sperm- and spermatocyte-specific proteins, VASA^12^, MIWI^13^ and GAPDH-S^14^ were not significantly changed in the mutation vs wild mouse testis (Fig. 3I).

Rimklb has a critical role in the process of spermatogenesis in seminiferous tubules; the mutation of Rimklb^A29del, L30V^ results in incomplete spermatozoa, which have been shown to be completely infertile.

### Rimklb mutation enhanced S6 phosphorylation

**Figure 4:**
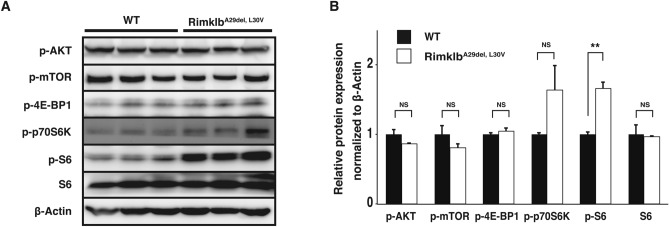
Hyperphosphorylation of ribosomal protein S6 in Rimklb^A29del, L30V^ mouse testis. (**A**) Western blot analysis of p-AKT, p-mTOR, p-4E-BP1, p-p70S6K, p-S6 and S6 in WT and Rimklb^A29del, L30V^ mouse testis at 12 weeks of age. β-Actin was used as a loading control except S6. Loading control of S6 was β-Actin as shown in Fig. 2D. (**B**) Graphic presentations show the expressions of p-AKT, p-mTOR, p-4E-BP1, p-p70S6K, p-S6 and S6. Data are presented as mean ± SEM; Student’s t-test; **p < 0.01, *NS* not significant. (n = 3).

**Figure 5:**
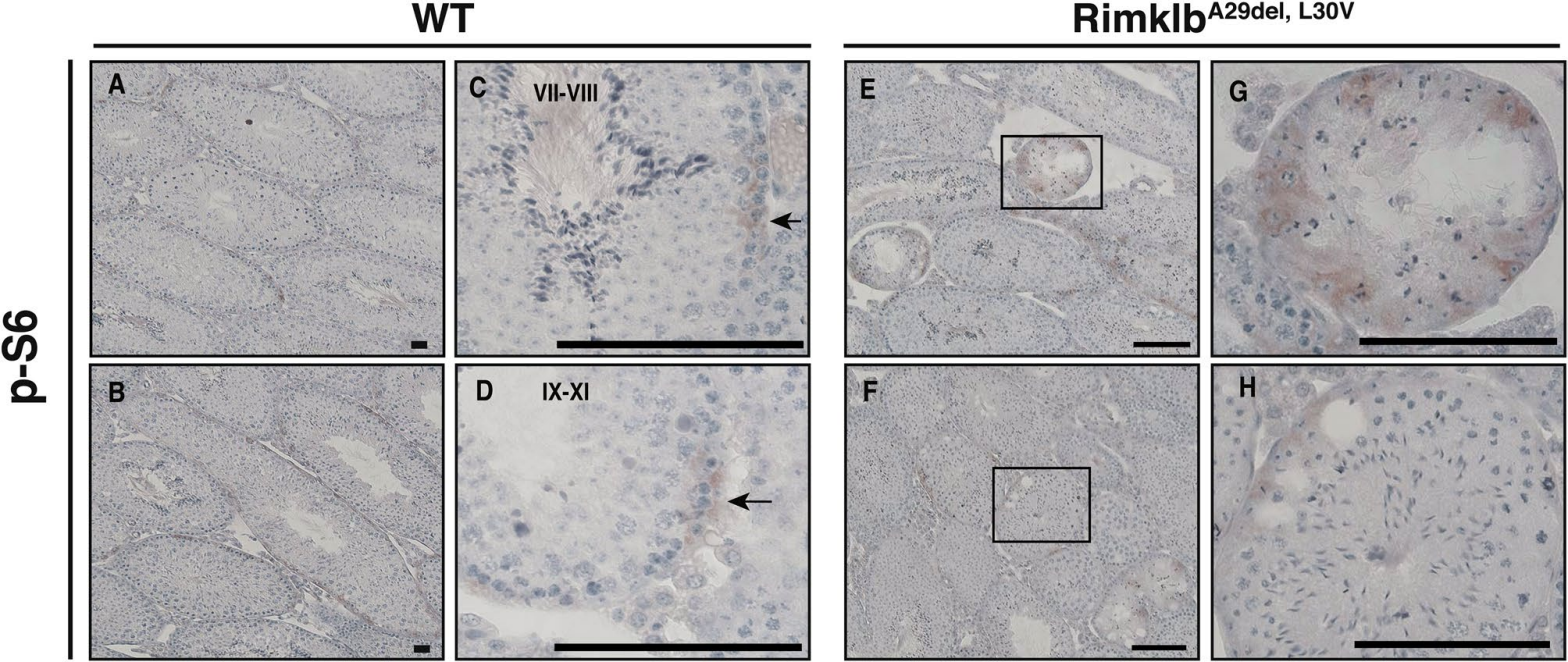
Hyperphosphorylation of ribosomal protein S6 in seminiferous tubules. (**A**,**B**) Immunohistochemistry of WT testis for p-S6. Seminiferous tubules stages are shown. (**C**,**D**) Enlarged images: immunohistochemistry of WT testis for p-S6. The black arrows indicate p-S6-positive cells. (**E**,**F**) Immunohistochemistry of Rimklb^A29del, L30V^ mouse testis for p-S6. (**G**,**H**) Enlarged images of the boxed area are shown. Scale bar = 100 μm.

Rimklb is a member of the rimK family, which modifies the ribosomal protein S6 in prokaryotes^15^. It has been reported that during spermatogenesis the mammalian S6 protein is downstream to the mTOR pathway, regulating the BTB and spermatogenesis^9^. To examine the relationship between Rimklb, mTOR and S6, we analyzed the effect of the Rimklb mutation on expression and phosphorylation of mTOR and S6 in the testis, comparing wild vs Rimklb^A29del, L30V^. We found that phosphorylated-S6 (p-S6) was obviously increased in the testes of Rimklb^A29del, L30V^ mutant mice. However, with p-AKT, p-mTOR, p-4E-BP1, p-p70S6K and S6, no significant changes could be observed (Fig. 4A,B). S6 is known to be the target protein of mTOR, which for spermatogenesis to occur is activated by phosphorylation via p70S6K^16^. To analyze the cell-specific expression of p-S6 in testes, we performed immunohistochemistry (IHC) using p-S6 antibody with hematoxylin staining. We found a weak p-S6 signal on the basement membrane side of the seminiferous tubules (Fig. 5A–D) for spermatocytes in stage VII-VIII or IX-XI germinal epithelia of WT testes. In addition, focal adhesion kinase (FAK) is known as a regulator of BTB dynamics in the testis^17^, the signals of which were detected near the basement membrane in the seminiferous tubule; p-S6 signals are also expressed in the seminiferous epithelium (Supplementary Fig. S2D). In the Rimklb^A29del, L30V^ mutant testis, stronger p-S6 protein expression was observed in the vacuoles of seminiferous tubules (Fig. 5E–H), and p-S6 positive tubules were obviously increased in the Rimklb^A29del, L30V^ mutant testis. Moreover, p-S6 positive tubules with vacuoles were distinctly increased in the Rimklb^A29del, L30V^ mutant testis (Table. 1).

**Table 1:** The number of p-S6-positive seminiferous tubules with increased vacuoles in the Rimklb^A29del, L30V^ mouse testis.

	Total tubules	Vacuoles	p-S6-positive	p-S6-positive with vacuoles
**Number of seminiferous tubules in testis**				
WT	149.0 ± 11.4	4.3 ± 0.5	30.7 ± 13.1	1.7 ± 1.3
Rimklb^A29del, L30V^	81.3 ± 18.9	34.0 ± 6.5	48.0 ± 11.0	23.0 ± 11.0

The total number of tubules, tubules with vacuoles, p-S6-positive tubules, p-S6-positive tubules with vacuoles in WT and Rimklb^A29del, L30V^ mouse testis at 12 weeks of age. Data are presented as mean ± SD.

## Discussion

In this study, we have shown that Rimklb^A29del, L30V^ males were completely infertile: both the ratios of average pups/litter and deliveries/plugs were zero on mating with Rimklb^A29del, L30V^ male mice. In addition, the International Mouse Phenotyping Consortium (https://www.mousephenotype.org) indicates that knockout (KO) of the Rimklb gene causes male infertility^18^. In Rimklb^A29del, L30V^ mutant mice, the testis weight was lower; sperm morphology analysis showed small, abnormally shaped heads; sperm counts were decreased; and when we tested the fertility of Rimklb^A29del, L30V^ male mice by mating them with wild C57BL/6 female for a two-month period (from eight weeks to 16 weeks of age) pregnancy failed to occur. IZUMO1, which plays an important role in sperm-egg fusion, was obviously reduced in the testes of Rimklb^A29del, L30V^ mutant male mice compared with wild mice. Furthermore, to determine how Rimklb is involved in spermatogenesis, we examined the expression of mTOR/S6 and the sperm-specific proteins that play crucial roles in spermatogenesis. We found that p-S6 was up-regulated around the vacuoles in seminiferous tubules within Rimklb^A29del, L30V^ testes.

Rimklb^A29del, L30V^ mutant mice have a deletion at amino acid 29 and the leucine at position 30 has been replaced with valine. Rimk has two ATP-binding sites: the lysine at position 158 and arginine at position 219 (UniProtKB-Q80WS1 (RIMKB_MOUSE)). One possibility is that A29del and L30V mutations in Rimklb affect the overall conformation and activity of Rimklb. We still need to conduct further experiments and analyses in order to elucidate the function of the Rimklb in spermatogenesis by Rimklb KO mice.

A previous study that found ß-CG in the adult rat testis^19^ is consistent with our finding that Rimklb is expressed in the mouse testis (Fig. 1). Rimklb^A29del, L30V^ male mice showed severe infertility including failure of spermatogenesis. One possible explanation is that Rimklb has a crucial role in the process of spermatogenesis through synthesis of ß-CG; however, we still have no direct evidence of the relationship between ß-CG and spermatogenesis.

Conversely, mammalian S6 is a key regulator in spermatogenesis^16^, especially in the mTOR/S6 pathway that is a critical signal transduction process in the Sertoli cell^11^. In this study, we have shown that the expression of mTOR, p-mTOR and S6 were unchanged in mutant vs wild mouse testis, however, p-S6 was obviously enhanced in the Rimklb^A29del, L30V^ mouse testis. Boyer et al. used conditional knockout mice (Mtor^flox/flox^; Amhr2^cre/+^ mice) to target mTOR in Sertoli cells, revealing the presence of large vacuoles in seminiferous tubules as well as severe male infertility. In addition, phosphorylation of RPS6 at S235/236 was upregulated in the testes of these mice^18^. This data indicates that down-regulation of mTOR in Sertoli cells inhibits spermatogenesis and leads to male infertility, resulting in enhanced phosphorylation of rps6. Their data are consistent with our observation for hyperphosphorylation of rpS6 and male infertility.

Interestingly, the p-S6 signal was observed in the vacuoles of seminiferous tubules, suggesting that the induction of p-S6 is possibly associated with seminiferous tubules and Sertoli cell function. Li et al. carried out experiments showing that the over-expressed and phosphorylated ribosomal protein S6 regulates the BTB, thereby negatively affecting spermatogenesis^9^, and rapamycin promotes autophagy and leads to suppression of spermatogenesis in the rat testis by inhibiting mTOR and p70S6 kinase^16^. Evidence has thus accumulated that p-S6 plays an important role in spermatogenesis.

Rimklb is expressed in Leydig cells, which are known to be involved in spermatogenesis by producing hormones such as testosterone. Rimklb^A29del, L30V^ mutant mice showed no difference in testosterone levels on comparing Rimklb^A29del, L30V^ mutants and wild male mice, suggesting that the mutation of Rimklb may not directly affect testosterone levels. A few studies have been conducted on S6 in Leydig cells: luteinizing hormone stimulated the phosphorylation of a 33,000 kDa protein in Leydig tumor cells^20^, and human chorionic gonadotropin (hCG) hormone enhanced p-S6 in primary cultures of porcine Leydig cells^21^. In this study, p-S6 expression was difficult to identify in Leydig cells, so the function of p-S6 in Leydig cells remains unclear. Further studies will be needed.

We have also shown that the expression of IZUMO1 was downregulated in Rimklb^A29del, L30V^ mutant testes. IZUMO1 is present in the acrosomal membrane and is known to play an important role during fertilization. Although it is not clear why IZUMO1 is decreased in the testes of Rimklb^A29del, L30V^ mutant mice, the functional changes putatively caused by the Rimklb^A29del, L30V^ mutation may suppress IZUMO1 expression.

Taken together, Rimklb is essential for spermatogenesis, and Rimklb is thought to be involved in all processes: spermatogenesis, spermatocyte-to-sperm differentiation, proliferation, and sperm fertilization. However, detailed mechanisms have not been elucidated, and further research must be conducted. Understanding the fine details of Rimklb may lead to elucidation of unknown mechanisms of male infertility.

## Methods

All experiments were performed in accordance with the relevant guidelines and regulations.

### Animal subjects

All mouse experiments were approved by the Kobe Gakuin Animal Experiment Committee (protocol No. A17-50) and the Animal Care and Use Committee of the National Institute of Quantum and Radiological Science and Technology (protocol No. 1610111 and 1610121). Mice were sacrificed using cervical dislocation performed by trained experimenters, or perfused and dissected under the three types of mixed anesthetic agents (0.3 mg/kg of medetomidine, 4.0 mg/kg of midazolam, and 5.0 mg/kg of butorphanol). Animal studies were conducted following the ARRIVE guidelines.

### Immunohistochemistry

The tissues were perfused and additionally fixed using Bouin fixation^22^ for 48 h. After fixation, the tissues were embedded in paraffin wax. Paraffin-embedded tissues were sliced to a thickness of six microns, attached to polylysine-coated slides, and dried at 40 °C overnight. The sliced tissues were deparaffinized using xylene, and immersed in ethanol and PBS. Antigens were retrieved in HistoVT One (Nacalai Tesque, Kyoto, Japan) by boiling for 20 min. In this study, tissue antigen signals were detected using the VECTASTAIN Elite ABC Kit (Vector Laboratories, Burlingame, CA, USA). In brief, for blocking, tissues were incubated in PBS containing normal goat serum for 20 min; the primary antibodies then used were anti-Rimklb (ab15783, Abcam, Cambridge, UK, Anti-RIMKB antibody N-terminal 1:100) and anti-Phospho-S6 Ribosomal Protein (#2211, Cell Signaling Technology, Danvers, MA, USA, Phospho-S6 Ribosomal Protein (Ser235/236) Antibody 1:400), applied overnight. Endogenous peroxidase was inactivated by 3% hydrogen peroxide for 15 min. The secondary antibody used was biotinyl-labeled anti rabbit antibody for 20 min; signal detection was performed by avidin-labeled peroxidase and DAB using the VECTASTAIN Elite ABC Kit. The sections (Phospho-S6 Ribosomal Protein) were counterstained with Mayer’s hematoxylin solution (FUJIFILM Wako Pure Chemical, Osaka, Japan). At least 50 effectively round seminiferous tubules were used for measurement of p-S6-positive tubules or p-S6-positive tubules with vacuoles. “P-S6-positive tubules” were counted if seminiferous tubules contained p-S6-positive cells, and “p-S6-positive tubules with vacuoles” were counted if seminiferous tubules contained positive cells and vacuoles.

### Generation of Rimklb mutant mouse

Rimklb mutant mice were generated using the CRISPR/Cas9 system and cytoplasmic microinjection of mouse embryos. Guide gRNAs (gRNAs) were designed to delete exon 2 of the Rimklb gene, and synthesized from 130 bp of chemically synthesized double-stranded DNA (gBlocks Gene Fragments, Integrated DNA Technologies, Coralville, IA, USA) that included the T7 promoter, the gRNA target sequence (AGAGATCTTACGAGCGTTGA) and the gRNA-scaffold sequence as a template using the MEGAshortscript T7 Transcription Kit (Life Technologies, Carlsbad, CA, USA) followed by RNA purification using a MEGAclear kit (Life Technologies). Embryo manipulation and microinjection were performed as previously described^23,24^. Briefly, MII-oocytes were collected from superovulated C57BL/6J females (aged 8–12 weeks, Japan SLC, Shizuoka, Japan), fertilized in vitro, and cultured in KSOM medium until use. Fertilized one-cell embryos underwent cytoplasmic microinjection with a mixture of recombinant Cas9 protein (50 ng/μl, NIPPON GENE, Tokyo, Japan) and two gRNAs (25 ng/μl). After the microinjection, the embryos were cultured in KSOM medium until the two-cell stage, and transferred to the oviduct of pseudopregnant ICR females (CLEA Japan, Tokyo, Japan) on the day of the vaginal plug (Day 0.5). Genomic DNA of offspring (F0 founders) was extracted from tail samples and used for genotyping. F0 founders harboring potential mutant alleles were bred with wild-type C57BL/6J mice, and mutations in the F1 generation were analyzed using the Guide-it Mutation Detection Kit (Takara Bio, Shiga, Japan). The mutant F2 females were crossed with wild BL/6 male mice; after mating mutant F3 mice with each other, TA cloning was used to obtain litter DNA for sequencing. One mouse line with deletion of three bases was chosen and used for this study.

### Genotyping

Mouse tails were lysed at 55 °C overnight, using lysis buffer containing Proteinase K (Sigma-Aldrich, St. Louis, MO, USA), and the lysate was directly used as a template for PCR. Genotyping of Rimklb mutant mice was performed using Ex Taq polymerase (Takara Bio) with a specific primer (Rimk1bCheckF: 5ʹ-CCTCATCCTCCTGTGCCTAA-3ʹ and Rimk1bCheckR: 5ʹ-GCACTCAGCTCTCCAGCTCT-3ʹ). PCR products were digested by the restriction enzyme Mwo I; the amplicon from the mutant allele was insensitive to Mwo I.

### Western blotting

The Western blotting shown in Fig. 1 was performed as previously described^25^. For the Western blotting of Fig. 4, mouse tissues were collected and lysed using a bead homogenizer (Mini bead-beater, WakenBtech, Kyoto, Japan) with 2.0 mm Zirconia beads (BioSpec Products, Bartlesville, OK, USA) in 1 mL cell-lysis buffer (#9803, Cell Signaling Technology) containing a proteinase inhibitor cocktail (#5871, Cell Signaling Technology). The lysates were centrifuged at 12,000 rpm for 20 min at 4 °C, and the supernatants were boiled in 4 × sample buffer (FUJIFILM Wako Pure Chemical) for 5 min. Protein samples were separated on a 10–20% gel (SuperSep Ace, FUJIFILM Wako Pure Chemical) and subsequently transferred to a PVDF membrane using the Turbo Transfer System (Bio-Rad, Hercules, CA, USA) with a seven-minute protocol. The membrane was blocked with Blocking One (Nacalai Tesque) for 30 min or 3% BSA (Rockland Immunochemicals, Inc., Pottstown, PA, USA) in T-BST for 1 h at room temperature, and incubated overnight at 4 °C with the following primary antibodies diluted 1:1000 in Blocking ONE: anti-phospho-S6 ribosomal protein (#4858, Cell Signaling Technology), anti-S6 ribosomal protein (#2317, Cell Signaling Technology), anti-phospho-mTOR (#5536, Cell Signaling Technology), anti-phospho-AKT (#4060, Cell Signaling Technology), anti-phospho-p70S6K (#9234 Cell Signaling Technology), anti-phospho-4E-BP1 (#2855, Cell Signaling Technology), horseradish peroxidase-conjugated anti-actin (A00730, GenScript, Piscataway, NJ, USA), anti-Miwi (#2079, Cell Signaling Technology), anti-GAPDH-S (13937-1-AP, Proteintech, Rosemont, IL, USA), and anti-Mouse Vasa Homologue (MVH/Vasa, ab13840, Abcam). Anti-IZUMO1 antibody was kindly provided by Dr. Masaru Okabe (Osaka University). Each membrane was incubated with an anti-mouse or rabbit IgG HRP-linked antibody (Cell Signaling Technology) as a secondary antibody diluted 1:10,000 in Blocking One at room temperature for 1 h. Chemiluminescence reactions were performed with Lightning Ultra (PerkinElmer, Waltham, MA, USA). The signals were detected using a ChemiDoc-It imaging system (Bio-Rad) with a BioChemi camera (UVP, Upland, CA, USA). Signal intensities were analyzed with NIH ImageJ (https://imagej.nih.gov/ij/). For some figures, unrelated lanes were cropped out. Full size images are provided in the Supplementary Figs. S3, S4.

### Histological staining

Testis sections were stained with hematoxylin and eosin after deparaffination. Slides were mounted and observed by microscopy (Model IX71, Olympus Corporation, Tokyo, Japan). At least 50 effectively round seminiferous tubules were used for measurement of vacuoles greater than ~ 30 μm in greatest diameter and located on or near the seminiferous tubule basement membrane, similar to previously reported methods^26^. Vacuoles values are all represented as the percentage of total tubules affected per total tubules counted.

### Testosterone assay

Serum testosterone analyses were performed using ELISA kits (Testosterone ELISA Kit, ADI-900-065, Enzo Life Sciences, Inc., Farmingdale, NY, USA). Serum was separated from all blood samples after centrifugation at 16,099×*g* for 15 min and frozen at − 90 °C for later hormonal analysis.

### Sperm counts and morphology

Sperm counts were performed as described by Wang^27^. The caudae epididymides of 12–13 week-old Rimklb^A29del, L30V^ and WT mice were collected in PBS, and minced using scalpel blades. After incubating for 15 min at 37 °C, sperm were diluted 1:4 in PBS and sperm counts determined on duplicate samples using a hemocytometer. Sperm were collected in the same manner and observed with a microscope. At least 250 sperm were observed for each experimental condition. Spermatozoa with round, thin, or bent heads were determined to be abnormal.

### Fertility test and IVF

Two C57BL/6J female mice and one male were kept in the same cage for two months until pregnancy resulted. Copulation was checked by examining for vaginal plugs every morning. IVF was performed as follows. The C57BL/6J female mice were injected intraperitoneally with pregnant mare serum gonadotropin (PMSG) (7.5 units, ASKA Pharmaceutical, Tokyo, Japan) and injected with human chorionic gonadotropin (hCG) (7.5 units, ASKA Pharmaceutical) 48 h later. MII-oocytes were collected from the ampulla of each oviduct of superovulated female mice 15 h after the injection of hCG. Spermatozoa were collected from the cauda epididymidis of three-month-old male mice and incubated in TYH medium for two hours. Capacitated spermatozoa were incubated in a drop with MII-oocytes, at a final concentration of 2 × 10^5^ sperm/mL. After incubation for 24 h, two-cell embryos were counted under a microscope.

## Supplementary Information

Supplementary Information 1.

Supplementary Video 1.
