# Supplementary material for: Rimklb mutation causes male infertility in mice
Source: Sci Rep. 2021 Feb 25;11:4604. doi: 10.1038/s41598-021-84105-z (PMC7907349; doi:10.1038/s41598-021-84105-z)
Supplement: Supplementary file 1 — Supplementary Information 1. [file 41598_2021_84105_MOESM1_ESM.docx]

Supplemental Information for:

Rimklb mutation causes male infertility in mice

Authors: Koji Maekura^1^, Satoshi Tsukamoto^2^, Michiko Hamada-Kanazawa^1^ , Masaoki Takano^1^

Affiliations: ^1^Laboratory of Molecular Cellular Biology, School of Pharmaceutical Sciences, Kobe Gakuin University, Japan

^2^Laboratory Animal and Genome Sciences Section, National Institute for Quantum and Radiological Science and Technology, Japan

**
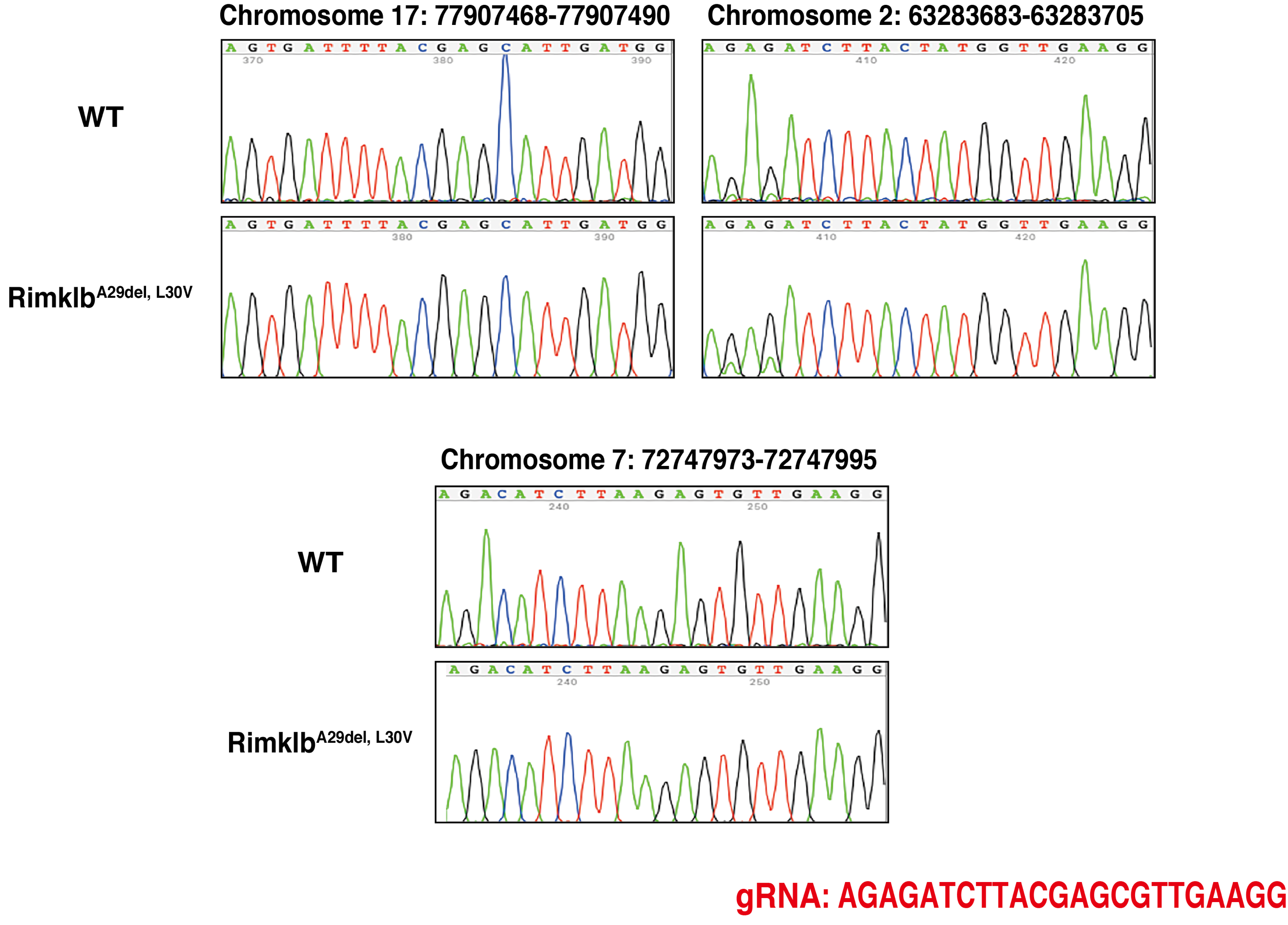
Supplementary Figure 1**

**The mutations did not occur in potential off-target sites.**

Potential off-target sites were searched using Off-spotter (<https://cm.jefferson.edu>) and CHOPCHOP (<https://chopchop.cbu.uib.no>). Three sites were selected and the region amplified by PCR. There were no genomic DNA sequences that differed from the Rimklb target site in one or two locations. The nucleotide sequence of PCR products was analyzed by direct sequencing. Chromosome numbers and regions are shown above the waveform data. There were no deletions or insertions at these sites.


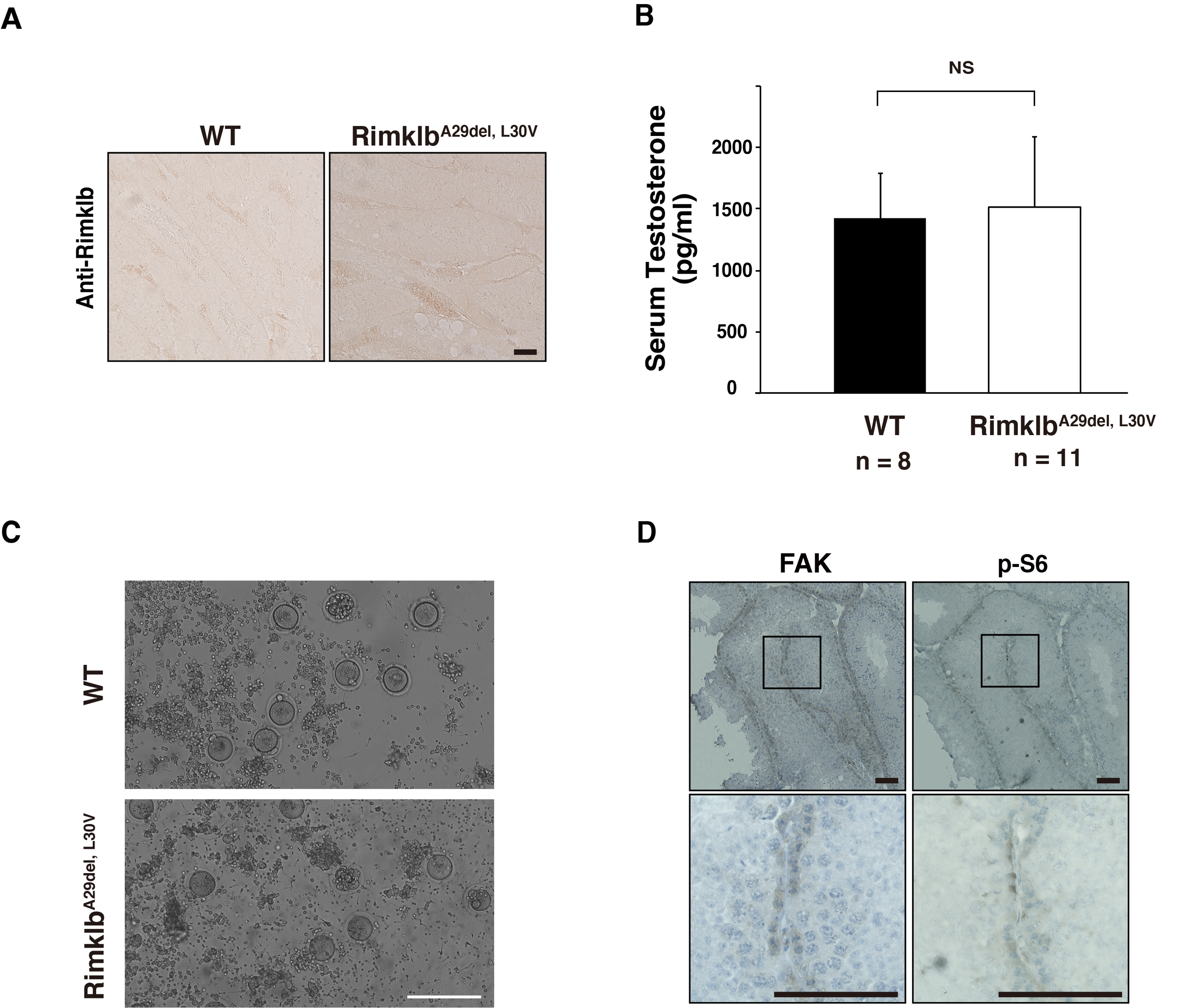


**Supplementary Figure 2**

**Testosterone assay and image of eggs, 6 h after insemination using *in vitro* fertilization.**

(A) Immunohistochemistry of WT and Rimklb^A29del, L30V^ mouse testis for Rimklb. Scale bar = 100 μm. (B) Serum testosterone levels of WT and Rimklb^A29del, L30V^ male mice. Data are presented as mean ± SEM; Student’s t-test; NS: Not Significant. (C) Representative images of eggs when oocytes were treated with wild type spermatozoa (upper) and Rimklb^A29del, L30V^ spermatozoa (bottom), 6 h after insemination. Scale bar = 200 μm. (D) Immunohistochemistry of WT testis for p-S6 and FAK. Enlarged images of the boxed area are shown (bottom). Scale bar = 100 μm.

**
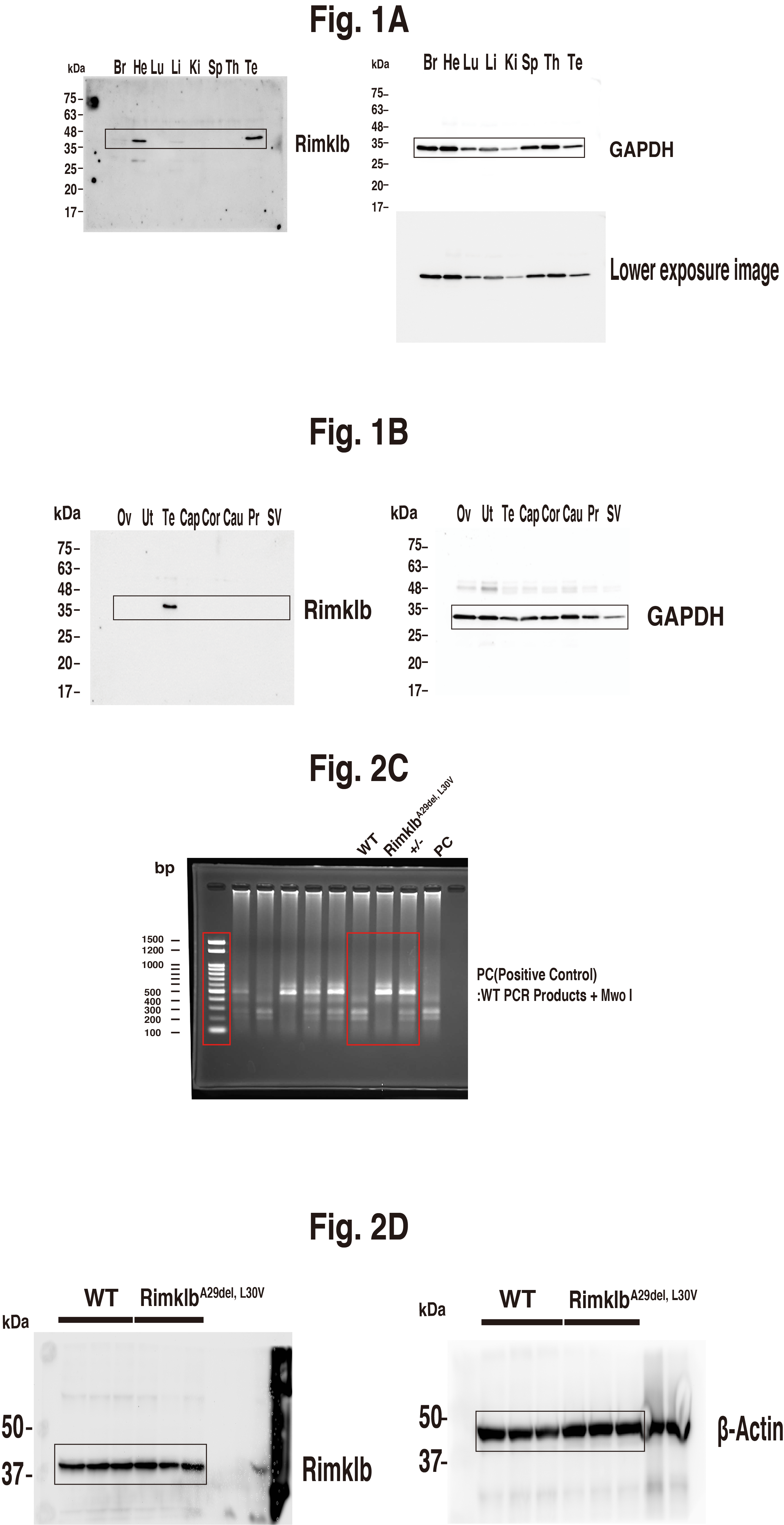
**

**Supplementary Figure 3**

**Full (uncropped) electrophoretic gel and blot images for main figures (Fig 1, 2).**

Molecular weight marker for DNA (bp) and protein (kDa) indicated. Black and red frames indicate cropped parts used in the main figures.


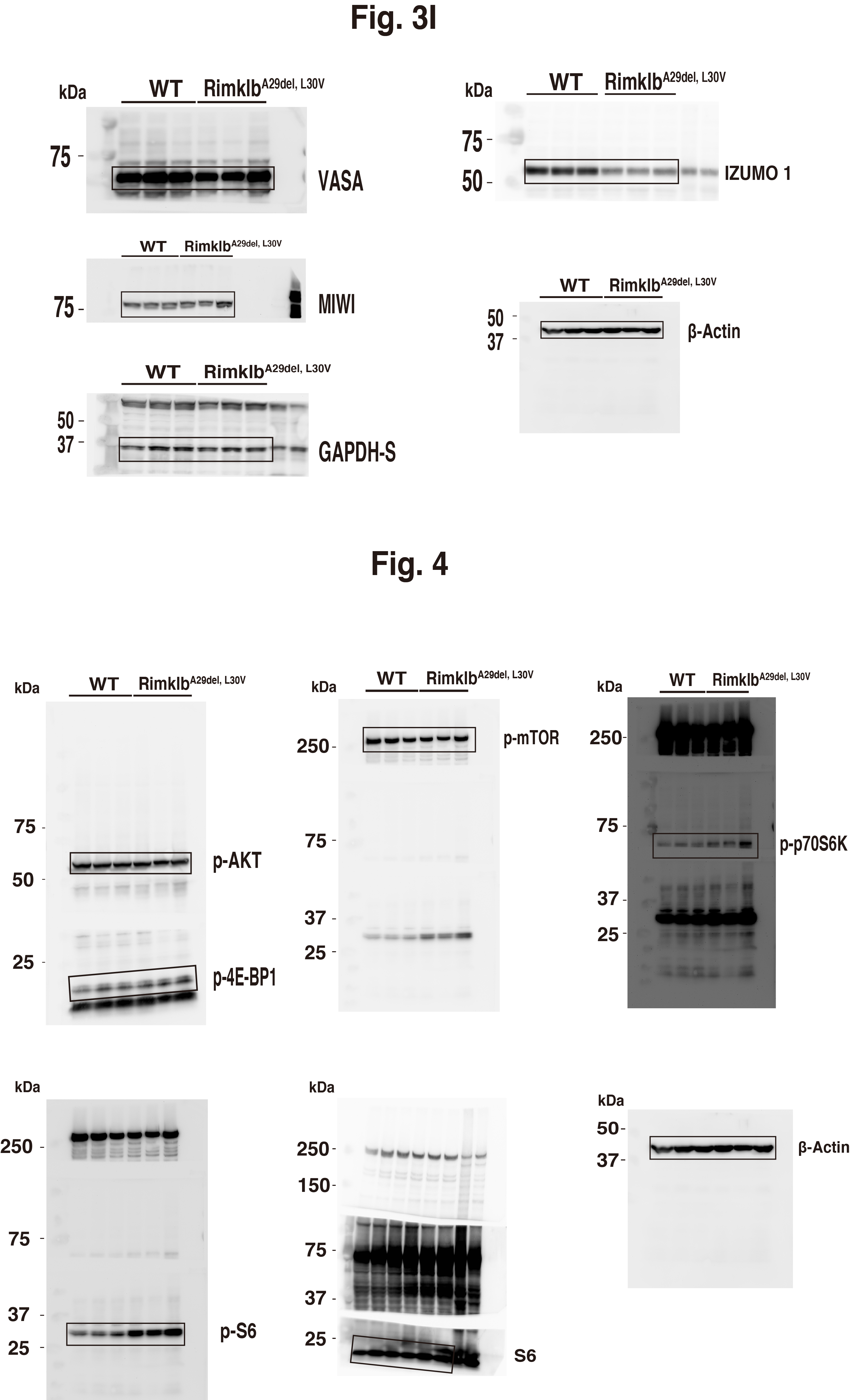


**Supplementary Figure 4**

**Full (uncropped) blot images for main figures (Fig 3, 4).**

Molecular weight marker for protein (kDa) indicated. Black frames indicate cropped parts used in the main figures.

**Supplementary Video Legend**

**Motility of WT and Rimklb^A29del, L30V^ spermatozoa; related to Figure 3**.

(A) WT and (B) Rimklb^A29del, L30V^ spermatozoa were allowed to disperse for 10 min pre-incubation in a 37°C chamber containing Human tubal fluid medium: video rate was 1/2 speed.
